# Poster Session I - A30 INVESTIGATING THE IMPACT OF PARKINSON’S DISEASE-ASSOCIATED GENE *PINK1* ON INTESTINAL HOMEOSTASIS AND RESPONSE TO INFECTION

**DOI:** 10.1093/jcag/gwaf042.030

**Published:** 2026-02-13

**Authors:** J Pei, S Recinto, A Kazanova, L Burns, A MacDonald, C Gavino, L Trudeau, M Desjardins, J Stratton, S Gruenheid

**Affiliations:** Microbiology and Immunology, McGill University, Montreal, QC, Canada; Microbiology and Immunology, McGill University, Montreal, QC, Canada; Microbiology and Immunology, McGill University, Montreal, QC, Canada; Microbiology and Immunology, McGill University, Montreal, QC, Canada; Microbiology and Immunology, McGill University, Montreal, QC, Canada; Microbiology and Immunology, McGill University, Montreal, QC, Canada; Universite de Montreal, Montreal, QC, Canada; Universite de Montreal, Montreal, QC, Canada; Microbiology and Immunology, McGill University, Montreal, QC, Canada; Microbiology and Immunology, McGill University, Montreal, QC, Canada

## Abstract

**Background:**

Intestinal epithelial cells (IECs) provide an essential physical barrier between luminal contents and host tissue. Dysregulation of IECs leads to barrier dysfunction, contributing to pathologies in both intestinal and extra-intestinal diseases. While Parkinson’s Disease (PD) is primarily a neurodegenerative disorder, increasing evidence links PD progression and gastrointestinal dysfunction. Our group developed a model to investigate the role of the gut in PD, demonstrating that mice with genetic ablation of the PD-associated gene *Pink1* exhibited motor phenotypes only when previously infected with gram-negative intestinal bacteria – *Citrobacter rodentium*. As *Pink1* is expressed in IECs and the colonic lamina propria, we hypothesize that PD-associated gene mutations directly affect the epithelium and impact early PD pathophysiology.

**Aims:**

1. Characterize transcriptional profiles of *Pink1* wild-type (WT) and knockout (KO) IECs at baseline and following *in vivo C. rodentium* infection.

2. Investigate *Pink1-*dependent epithelial responses using isolated *in vitro* and *ex vivo* systems.

**Methods:**

Single-cell RNA sequencing (scRNAseq) was performed on colonic IECs isolated from *Pink1* WT and KO mice, at steady state and following *in vivo C. rodentium* infection. Mice were sacrificed at day 7 and day 14 post-infection to capture transcriptional changes across IEC lineages. To further explore pathways identified by scRNAseq, *ex vivo* colonoids derived from *Pink1* WT and KO crypts, as well as *Pink1* KO CMT-93 epithelial cell line with WT control, will be used in complementary validation assays. We will also complete colonic epithelium-T cell co-culture experiments to evaluate MHC class I-restricted antigen presentation.

**Results:**

At baseline, our data revealed upregulation of interferon (IFN)-signaling genes (ISGs) in *Pink1* KO colonocytes compared to WT. During infection, *Pink1* KO absorptive progenitors showed significant alterations in ISGs and actin-cytoskeleton-related GO term pathways compared to WT. Additionally, *Pink1* KO stem cells, transit-amplifying cells, absorptive progenitors, and colonocytes demonstrated increase in antigen presentation and processing-related pathways. Further *in vivo* and *in vitro* work will be completed to validate these findings.

**Conclusions:**

Using *in vivo* and *in vitro* models encompassing PD-genetic susceptibility and environmental stimuli, we identified dysregulation in *Pink1* KO epithelium, suggesting altered inflammatory responses at baseline and infection. By investigating PD-associated genes in IECs, we will contribute to better understanding the role of the intestine in PD initiation, progression, and pathogenesis.

**Funding Agencies:**

CIHR, NoneTRIANGLE

